# The Book World of Medicine and Science

**Published:** 1897-01-02

**Authors:** 


### The Book World of Medicine and Science.

The Treatment of Phthisis. By Arthur Ransome, M.D., M.A., F.R.S. 8vo., pp. 237. (London: Smith, Elder, and Co. 1896.)

The long experience, together with the persistent and scientific investigations of the author on the causes and treatment of phthisis render this small work peculiarly acceptable and valuable to all practitioners of medicine. Dr. Ransome makes no attempt to produce here a complete treatise on the subject in hand, and states in his preface that he has thought it best to confine his observations chiefly to his own experience, and to record for the most part only such methods as have been used under his own eyes. But the author's work has been conducted in no narrow groove, and his experience is wide, so that the monograph before us forms a most reliable guide to those seeking assistance in dealing practically with the devastations of pulmonary tuberculosis. His moderate language, breathing in every line a ripe experience and deep research, will give confidence afresh to all who have been disappointed, as all of us must be at times, with the repeated failures attending the treatment of this disease. In the opening chapter on the curability of phthisis the author endorses Dr. Harris' statement that about 39 per cent. of all non-tubercular post-mortem examination cases over twenty years of age present signs of involuted tubercle. In the curability of phthisis he is a firm believer. In his following chapter, on the ætiology of phthisis, while the bacillus is held to be the essential factor, great stress is laid on the all-important contributing factors which permit the bacillus to gain a footing and form "a colony." The author rightly emphasises the importance of recognising the many safeguards against invasion which protect the healthy, and dilates on the causes of these safeguards breaking down in the infected. Perhaps one of the characteristic features of the book is the clear demonstration of the methods by which infection may be avoided, and of the general hygienic principles which are the only successful means by which prophylaxis may be accomplished. But the chapters on treatment are up to date, enlightening, and encouraging.

The Practice of Midwifery. A Guide for Practitioners and Students. By D. Lloyd Roberts, M.D., F.R.C.P. Fourth edition, foolscap octavo, pp. 585, with two coloured plates and 226 woodcuts. Price 10s. 6d. (London: J. and A. Churchill. 1896.)

This is the fourth edition of Dr. Lloyd Roberts's "Practice of Midwifery." It is larger and more profusely illustrated than the previous edition. The anatomy of the female pelvis, the mammary glands, and the female generative organs is first fully described. There is then a chapter on ovulation and menstruation and one on the development of the embryo. It may be a matter of opinion as to whether the student should get his knowledge of embryology from a special treatise or have it provided for him in his midwifery manual, but the subject is here fully described and well illustrated; we think, however, that a student who had no previous acquaintance with the subject would have some difficulty in understanding the description given of the process by which the embryo becomes separated from the rest of the blastodermic vesicle. A diagrammatic or semi-diagrammatic cut would make this much plainer. The various pelvic deformities are well described and illustrated, but there does not appear to be any description of the mechanism of labour in the various types of deformed pelvis.

There are two good coloured plates of different stages of the corpus luteum of menstruation and pregnancy. The author has added a new chapter on the use of antiseptics in midwifery; full directions are given for the antiseptic conduct of labour, and stress is laid upon the care of the nipples as a preventative of mastitis and upon a dry dressing for the umbilical cord. It is to be feared that in practice the antiseptic care of the child and mother has not received the attention it deserves.

In the chapter on post-partum hæmorrhage the treatment for the average case is well and clearly described, and the reader is not tormented by a choice of too numerous remedies. We think, however, that the student would be both better able and more likely to treat the collapse arising from it by intra-venous transfusion if told that all he needed besides scalpel and catgut was some hot water, a clean Higginson's syringe, a small glass or metal cannula with a piece of drainage tubing, and some common salt. This apparatus has sufficed for the successful treatment of hæmorrhage in various surgical conditions, and there is no reason why it should not be equally successful in obstetrical practice. Post-partum hæmorrhage is nearly always a matter of emergency, and as such demands prompt treatment by the simplest possible means; arm to arm transfusion can seldom be carried out, is more difficult to perform, and takes up more time; blood for mediate transfusion is not so available; water as hot as the hand can bear and common table salt can, however, be obtained in the poorest house. A chapter on the use and choice of anæsthetics is added at the end.

The book is well printed and illustrated, its matter is brought well up to date, and it can be confidently recommended as a reliable guide either to the practitioner or the student.

